# The Combination of Niacinamide, Vitamin C, and PDRN Mitigates Melanogenesis by Modulating Nicotinamide Nucleotide Transhydrogenase

**DOI:** 10.3390/molecules27154923

**Published:** 2022-08-02

**Authors:** Hyun Jun Park, Kyung-A Byun, Seyeon Oh, Hyoung Moon Kim, Moon Suk Chung, Kuk Hui Son, Kyunghee Byun

**Affiliations:** 1 Maylin Anti-Aging Center Apgujeong, Seoul 06005, Korea; parmani@naver.com; 2 Department of Anatomy & Cell Biology, College of Medicine, Gachon University, Incheon 21936, Korea; kabyun95@gmail.com (K.-A.B.); drmac12@me.com (H.M.K.); 3 Functional Cellular Networks Laboratory, Graduate School and Lee Gil Ya Cancer and Diabetes Institute, College of Medicine, Gachon University, Incheon 21999, Korea; seyeon8965@gmail.com; 4 I’ll Global Co., Inc., Seoul 06532, Korea; sugi0821@empas.com; 5 Department of Thoracic and Cardiovascular Surgery, Gil Medical Center, Gachon University, Incheon 21565, Korea

**Keywords:** melanogenesis, niacinamide, nicotinamide nucleotide transhydrogenase, oxidative stress, vitamin C

## Abstract

Nicotinamide nucleotide transhydrogenase (NNT) is involved in decreasing melanogenesis through tyrosinase degradation induced by cellular redox changes. Nicotinamide is a component of coenzymes, such as NAD^+^, NADH, NADP^+^, and NADPH, and its levels are modulated by NNT. Vitamin C and polydeoxyribonucleotide (PDRN) are also known to decrease skin pigmentation. We evaluated whether a mixture of nicotinamide, vitamin C, and PDRN (NVP-mix) decreased melanogenesis by modulating mitochondrial oxidative stress and NNT expression in UV-B-irradiated animals and in an in vitro model of melanocytes treated with conditioned media (CM) from UV-B-irradiated keratinocytes. The expression of NNT, GSH/GSSG, and NADPH/NADP^+^ in UV-B-irradiated animal skin was significantly decreased by UV-B radiation but increased by NVP-mix treatment. The expression of NNT, GSH/GSSG, and NADPH/NADP^+^ ratios decreased in melanocytes after CM treatment, although they increased after NVP-mix administration. In NNT-silenced melanocytes, the GSH/GSSG and NADPH/NADP^+^ ratios were further decreased by CM compared with normal melanocytes. NVP-mix decreased melanogenesis signals, such as MC1R, MITF, TYRP1, and TYRP2, and decreased melanosome transfer-related signals, such as RAB32 and RAB27A, in UV-B-irradiated animal skin. NVP-mix also decreased MC1R, MITF, TYRP1, TYRP2, RAB32, and RAB27A in melanocytes treated with CM from UV-irradiated keratinocytes. The expression of MC1R and MITF in melanocytes after CM treatment was unchanged by NNT silencing. However, the expression of TYRP1, TYRP2, RAB32, and RAB27A increased in NNT-silenced melanocytes after CM treatment. NVP-mix also decreased tyrosinase activity and melanin content in UV-B-irradiated animal skin and CM-treated melanocytes. In conclusion, NVP-mix decreased mitochondrial oxidative stress by increasing NNT expression and decreased melanogenesis by decreasing MC1R/MITF, tyrosinase, TYRP1, and TYRP2.

## 1. Introduction

Melanin is generated in melanocytes from indole compounds that are synthesized from the amino acid tyrosine [1]. There are two types of melanin: soluble yellow to reddish pheomelanin and insoluble black to brown eumelanin. The ratio between pheomelanin and eumelanin determines skin and hair color [1]. Both eumelanin and pheomelanin are generated from the same precursor dopaquinone. The enzyme tyrosinase induces the oxidization of tyrosine to make dihydroxyphenylalanine (DOPA) and generate dopaquinone [1].

The generation of eumelanin is initiated by the production of cycloDOPA from dopaquinone. Then, cycloDOPA rapidly changes into DOPAchrome and DOPA by redox exchange [2]. DOPAchrome is spontaneously decarboxylated into 5,6-dihydroxyindole (DHI) [3]. Moreover, DOPAchrome also generates 5,6-dihydroxyindole-2-carboxylic acid (DHICA) by the action of tyrosinase-related protein 2 (TYRP2) [3]. Both DHI and DHICA are transformed into eumelanin by further oxidization and polymerization via tyrosinase or tyrosinase-related protein 1 (TYRP1) [3].

Eumelanin has a structure of fibrillar melanosomes and expresses a structural matrix protein, namely premelanosomal protein 17 (PMEL17), which is an amyloid protein and a material for generation of internal fibrils [4]. This fibril structure is not present in pheomelanin [4]. Pheomelanin production starts with the reductive addition of cysteine to dopaquinone [5,6,7]. On the other hand, the generation of eumelanin is determined by the activity of tyrosinase, TYRP1, and TYRP2, whereas the fabrication of pheomelanin is determined by tyrosinase and the presence of cysteine [8].

Melanin is stored in melanosomes, which are lysosome-related organelles [9]. Melanosome maturation occurs in four stages. Melanosomes in stages I and II lack melanin deposition; however, melanosomes in stages III and IV contain melanin [10,11]. In stage I, the melanosome structure is similar to that of endosomes, as both have intraluminal vesicles. In stage II, PMEL fibrils are organized as a parallel sheet, and the shape of the melanosomes changes to ellipsoid, but pigmentation is still absent [10,11]. These fibrils act as a matrix for melanin synthesis, which is initiated from stage III melanosomes [10,11]. In stage III, tyrosinase or TYRP1 is transferred to the melanosomes by Ras-like GTPases (RABs) 32 and 38 [12]. In stage IV, melanin is sequestered on the fibrils, completely masking the fibrils [10,11]. Mature melanosomes are transported to the dendritic tips of melanocytes by Rab27, which is the main regulator of intracellular membrane trafficking [13].

From the dendritic tips of melanocytes, the melanosomes are transferred to keratinocytes to protect the skin from UV radiation [14], as excessive UV radiation leads to skin hyperpigmentation. UV radiation upregulates proopiomelanocortin (POMC), which increases α-melanocyte-stimulating hormone (α-MSH) in keratinocytes [15]. α-MSH binds to melanocortin 1 receptor (MC1R) in melanocytes, which eventually causes upregulated transcription of microphthalmia-associated transcription factor (MITF) [16]. MITF acts as a main regulator of melanogenesis by increasing the amounts of melanogenic enzymes such as tyrosinase, TYRP1, and, TYRP2 [17,18]. It is also known that MITF upregulates Rab32 and Rab27A [19,20].

Moreover, nicotinamide nucleotide transhydrogenase (NNT) was recently shown to be involved in melanin synthesis in the skin [21]. NNT exists in the inner mitochondrial membrane and transports H^+^ to the inside of the mitochondria [22,23]. Coupled with H^+^ translocation, NNT transfers hydride (H^-^) from reduced nicotinamide adenine dinucleotide (NADH) to nicotinamide adenine dinucleotide phosphate (NADP^+^) [22,23]. Through these reactions, NNT increases the ratio of reduced nicotinamide adenine dinucleotide phosphate (NADPH) to NADP^+^ (NADPH/NADP^+^) in the mitochondrial matrix [24]. Moreover, NNT generates NADPH using NADH as an electron donor [25]. NADPH plays an essential role in the antioxidant system by maintaining levels of reduced glutathione (GSH) and thioredoxin (TRX) [26,27,28]. NADPH is used as a cofactor for GSH reductase, which converts oxidized glutathione (GSSG) into GSH [29,30]. The conversion of GSSG to GSH is also induced by glutathione peroxidase (GPX). GPX uses hydrogen peroxide, which is generated from superoxide anions by the action of superoxide dismutase (SOD) to protect cells against reactive oxygen species (ROS)-induced injury [22,23,31,32].

NNT induces cellular redox changes that lead to tyrosinase degradation and melanosome maturation modulation to decrease eumelanin levels [21]. By removing NNT from human melanoma cells, melanin synthesis increases [21]. In contrast, overexpression of NNT decreases the synthesis of eumelanin, which is accompanied by an increase in the NADPH/NADP^+^ and GSH/GSSG ratios [21].

Nicotinamide (niacinamide) is a component of coenzymes such as nicotinamide adenine dinucleotide (NAD^+^), NADH, NADP^+^, and NADPH [33,34]. External supplementation with nicotinamide increases the functions of these coenzymes and leads to decreases in ROS-related injuries in cells [35]. Nicotinamide was reported to increase the lifespan of human fibroblasts by decreasing the production of mitochondrial ROS [36].

Moreover, nicotinamide has been used as a material in various cosmetics. Nicotinamide attenuates UV light-induced DNA damage in epidermal melanocytes [37] and shows a skin whitening effect by decreasing both tyrosinase activity and melanosome transfer from melanocytes to keratinocytes [38,39,40].

Even though nicotinamide is a precursor of various coenzymes, such as NADH and NADP^+^, and nicotinamide has been used as a whitening agent in cosmetics, it has not been revealed whether nicotinamide decreases melanogenesis by modulating NNT. Vitamin C is a well-known antioxidant that protects against UV-induced oxidative damage by scavenging free radicals and ROS [41,42]. Polydeoxyribonucleotide (PDRN) contains deoxyribonucleotide polymers that are extracted from the sperm of trout or salmon [43]. PDRN causes the downregulation of tyrosinase, TYRP1, and MITF and shows an anti-melanogenesis effect [44]. Previously, our group reported that a mixture of nicotinamide, vitamin C, and PDRN decreased destruction of collagen and elastin fiber by decreasing metalloproteinases via modulating nuclear factor erythroid 2-like 2 and heme oxygenase-1 which reduced expression of NADPH oxidase [45].

Taken all together, nicotinamide, vitamin C, and PDRN individually show anti-melanogenic effects; however, the result of treatment with a mixture of these compounds on decreasing skin pigmentation via NNT has not been fully determined.

In this study, we evaluated whether a mixture of nicotinamide, vitamin C, and PDRN (NVP-mix) decreased skin pigmentation by modulating NNT in UV-B-irradiated animal skin.

## 2. Results

### 2.1. NVP-Mix Increased NNT Expression and Decreased Mitochondrial Oxidative Stress in UV-B-Irradiated Animal Skin

**Figure 1:**
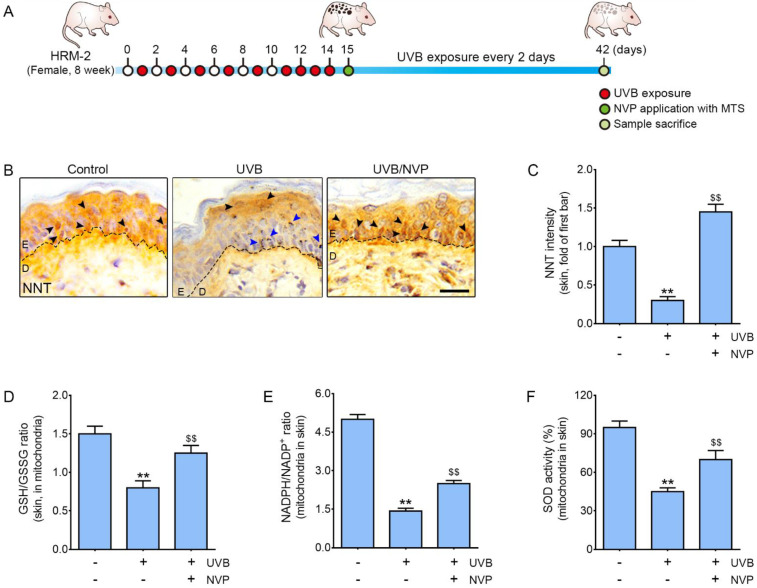
Regulation of the expression of NNT, the GSH/GSSG ratio, the NADPH/NADP^+^ ratio, and SOD activity after UV-irradiated animal skin was treated with NVP-mix. (**A**) Schematic diagram of the animal experiment used in this study. (**B**) NNT expression was determined by immunohistochemical analysis of the epidermal tissues of UV-irradiated mice (scale bar = 100 µm). The black arrows indicate NNT-positive cells, and blue arrows indicate melanin. (**C**) The intensity of NNT in the epidermis of UV-irradiated animals (image B) was quantified using ImageJ software. (**D**–**F**) The GSH/GSSG ratio in the mitochondria (**D**), NADPH/NADP^+^ ratio in the mitochondria (**E**), and SOD activity in the mitochondria (**F**) were measured in UV-irradiated animal skin. Data are presented as the mean ± standard deviation; ** *p* < 0.01 second bar vs. first bar; $$ *p* < 0.01 vs. second bar (Mann–Whitney U test). D, dermis; E, epidermis; GSH, glutathione; GSSG, oxidized glutathione; MTS, microneedle therapy system; NADP^+^, nicotinamide adenine dinucleotide phosphate; NNT, nicotinamide nucleotide transhydrogenase; NVP, niacinamide + vitamin C + polydeoxyribonucleotide; SOD, superoxide dismutase; UV, ultraviolet.

First, we evaluated whether NVP-mix modulated NNT in UV-B-irradiated animal skin. The expression levels of NNT in the epidermal tissues of the control animals (control group), UV-B-irradiated animals (UV group), and NVP-mix-applied UV-B-irradiated animals (UV/NVP group) were compared. Notably, the mice in the UV group and UV/NVP group were exposed to UV-B irradiation every 2 days until the end of the experiment. Fifteen days after the start of the experiment, the UV/NVP group was administered 50 µL of NVP-mix per square centimeter of skin at 7-day intervals via a microneedle therapy system (MTS). The control and UV groups were administered distilled water via the MTS (Figure 1A).

The expression of NNT in the epidermises of the animals in the UV group was significantly lower than that in the control group, whereas the expression of NNT in the UV/NVP group was significantly higher than that in the UV group (Figure 1B,C).

To evaluate the modulation of mitochondrial oxidative stress caused by NVP-mix in animal skin, mitochondria were isolated from the skin with a mitochondria isolation kit for tissue. The GSH/GSSG ratio in the mitochondria from the skins of the animals in the UV group was significantly lower than that in the control and UV/NVP groups (Figure 1D). Additionally, the mitochondrial NADPH/NADP^+^ ratio in the skins of the mice in the UV group was significantly lower than that found in the control and UV/NVP groups (Figure 1E). Moreover, the SOD activity in the mitochondria from the skins of the animals in the UV group was significantly lower than that in the control and UV/NVP groups (Figure 1F).

Thus, NVP-mix increased the expression of NNT, the GSH/GSSG ratio, NADPH/NADP^+^ ratio, and SOD activity, all of which were decreased by UV-B irradiation.

### 2.2. NVP-Mix Increased the Expression of NNT and Decreased UV-B-Induced Mitochondrial Oxidative Stress in Human Melanocytes

We next evaluated whether NVP-mix decreased mitochondrial oxidative stress by modulating NNT with an in vitro model. NVP-mix was applied to animal skin via the MTS. Here, we assumed that NVP-mix first affected the keratinocytes in the epidermis and then the effective factors secreted from the keratinocytes modulated the melanocytes. Human keratinocytes were exposed to UV-B irradiation for 5 min, and then PBS or NVP-mix was administered. The supernatant from the PBS-treated UV-B-irradiated keratinocytes was administered to normal melanocytes (UV-CM) or NNT-silenced melanocytes (*si*NNT/UV-CM) and the supernatant from the NVP-mix-treated UV-B-irradiated keratinocytes was administered to normal melanocytes (UV-NVP-CM) or NNT-silenced melanocytes (*si*NNT/UV-NVP-CM) (Figure S1).

**Figure 2:**
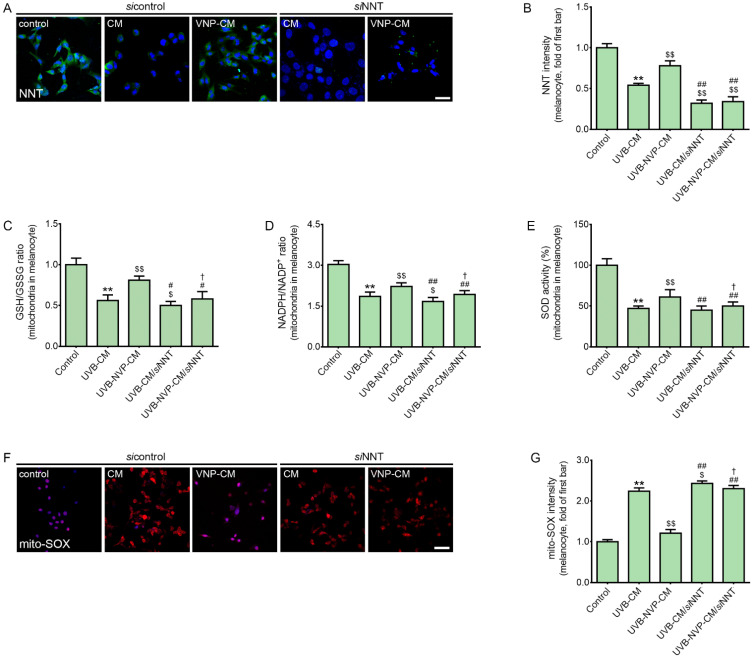
Regulation of the expression of NNT, the GSH/GSSG ratio, the NADPH/NADP^+^ ratio, SOD activity, and MitoSOX caused by NVP-mix treatment in melanocytes. (**A**) NNT expression in CM-treated melanocytes was determined by immunocytochemistry (scale bar = 50 µm) (green: NNT; blue: DAPI for nuclear staining). CM was obtained from UV-irradiated keratinocyte cultures treated with PBS or NVP-mix. (**B**) The intensity of NNT in the cells (image A) was quantified using Zen 2009 software. (**C**–**E**) The GSH/GSSG ratio in the mitochondria (**C**), NADPH/NADP^+^ ratio in the mitochondria (**D**), and SOD activity in the mitochondria (**E**) were measured with assay kits after isolation of the mitochondria from CM-treated melanocytes. (**F**) Mitochondrial peroxide levels were quantified with the fluorescent dye MitoSOX Red by immunofluorescence staining (scale bar = 50 µm) (red: mito-SOX; blue: DAPI for nuclear staining). (**G**) The intensity of mito-SOX (image F) was quantified using Zen 2009 software. Data are presented as the mean ± standard deviation; ** *p* < 0.01 second bar vs. first bar; $ *p* < 0.05, $$ *p* < 0.01 vs. second bar; # *p* < 0.05, ## *p* < 0.01 vs. third bar, † *p* < 0.05 vs. fourth bar (Mann–Whitney U test). CM, conditioned medium; DAPI, 4′,6-diamidino-2-phenylindole; GSH, glutathione; GSSG, oxidized glutathione; NADP^+^, nicotinamide adenine dinucleotide phosphate; NNT, nicotinamide nucleotide transhydrogenase; NVP, niacinamide + vitamin C + polydeoxyribonucleotide; SOD, superoxide dismutase; UV, ultraviolet.

The expression of NNT in the melanocytes, which was evaluated by NNT staining, decreased significantly after treatment with UV-CM (Figure 2A,B). However, NNT expression was significantly increased by UV-NVP-CM treatment. The expression of NNT in the UV-CM group was significantly higher than that in the *si*NNT/UV-CM group. This result shows that the decrease in NNT expression induced by UV-CM was significantly higher in NNT-silenced melanocytes than in normal melanocytes. The expression of NNT in the *si*NNT/UV-NVP-CM group was not significantly different from that in the *si*NNT/UV-CM group. Therefore, in NNT-silenced melanocytes, NVP-mix did not increase NNT expression.

The GSH/GSSG and NADPH/NADP^+^ ratios and SOD activity were evaluated after melanocyte mitochondrial isolation. The GSH/GSSG ratio was significantly decreased by UV-CM treatment and significantly increased by NVP-mix treatment (Figure 2C). The GSH/GSSG ratio in the UV-CM group was significantly higher than that in the *si*NNT/UV-CM group. This result showed that the decrease in the GSH/GSSG ratio induced by UV-CM was significantly higher in NNT-silenced melanocytes than in normal melanocytes. The GSH/GSSG ratio in the *si*NNT/UV-NVP-CM group was not significantly different from that in the *si*NNT/UV-CM group. However, the GSH/GSSG ratio in the *si*NNT/UV-NVP-CM group was significantly higher than that in the UV-NVP-CM group.

The NADPH/NADP^+^ ratio significantly decreased after UV-CM treatment and significantly increased after NVP-mix treatment (Figure 2D). The NADPH/NADP^+^ ratio in the UV-CM group was significantly higher than that in the *si*NNT/UV-CM group. This result showed that the decrease in the NADPH/NADP^+^ ratio induced by UV-CM was significantly higher in NNT-silenced melanocytes than in normal melanocytes. The NADPH/NADP^+^ ratio in the *si*NNT/UV-NVP-CM group was significantly higher than that in the *si*NNT/UV-CM group and significantly lower than that in the UV-NVP-CM group. This result indicated that the NADPH/NADP^+^ ratio was increased by NVP-mix treatment in NNT-silenced melanocytes; however, this effect was reduced in NNT-silenced melanocytes compared with normal melanocytes.

SOD activity was significantly decreased by UV-CM treatment and significantly increased by NVP-mix treatment (Figure 2E). Moreover, SOD activity after UV-CM treatment was not significantly different between normal and NNT-silenced melanocytes. SOD activity increased after administration of NVP-mix to NNT-silenced melanocytes; however, this increase was less pronounced compared with normal melanocytes.

The MitoSOX assay is frequently used to detect mitochondrial superoxide or ROS [46]. MitoSOX assays showed that oxidative stress in the mitochondria of melanocytes increased after UV-CM treatment; additionally, oxidative stress decreased with UV-NVP-CM administration (Figure 2F,G). Oxidative stress in the UV-CM group was significantly lower than that in the *si*NNT/UV-CM group. This result showed that the increase in oxidative stress induced by UV-CM was higher in NNT-silenced melanocytes than in normal melanocytes. Oxidative stress in the *si*NNT/UV-NVP-CM group was lower than that in the *si*NNT/UV-CM group; nonetheless, oxidative stress in the *si*NNT/UV-NVP-CM group was higher than that in the UV-NVP-CM group. This result indicated that NVP-mix could decrease oxidative stress in NNT-silenced melanocytes, but this effect was lower than that in normal melanocytes. These results suggested that NVP-mix decreased mitochondrial oxidative stress induced by UV-B irradiation via NNT upregulation.

### 2.3. NVP-Mix Decreased the Expression of Melanogenesis Signaling Pathway Components in UV-B-Irradiated Animal Skin

The expression levels of MC1R and MITF in the UV group were significantly higher than those in the control or UV/NVP groups (Figure S2A,B), as were the expression levels of TYRP1 and TYRP2 (Figure S2C,D) and RAB32 and RAB27A (Figure S2E,F). These results suggested that NVP-mix decreased well-known melanogenesis signaling pathways, such as MC1R, MITF, TYRP1, TYRP2, RAB32, and RAB27A.

### 2.4. NVP-Mix Decreased Melanogenesis-Related Signaling Pathways in Human Melanocytes

**Figure 3:**
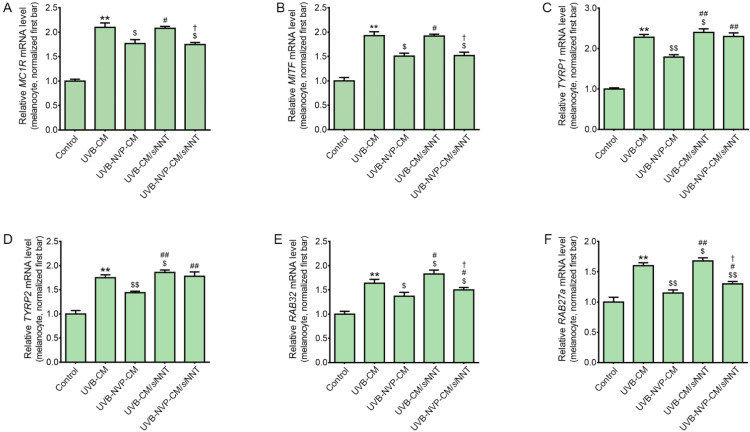
Regulation of the expression of MC1R, MITF, TYRP1, TYRP2, RAB32, and RAB27A after NVP-mix treatment to melanocytes. (**A**–**F**) The mRNA expression levels of MC1R (**A**), MITF (**B**), TYRP1 (**C**), TYRP2 (**D**), RAB32 (**E**), and RAB27A (**F**) were determined by quantitative real-time polymerase chain reaction in CM-treated melanocytes. CM was obtained from UV-irradiated keratinocyte cultures treated with PBS or NVP-mix. The mRNA levels were normalized to that of Actb and are expressed relative to the corresponding level in the control group. Data are presented as the mean ± standard deviation; ** *p* < 0.01 second bar vs. first bar; $ *p* < 0.05, $$ *p* < 0.01 vs. second bar; # *p* < 0.05, ## *p* < 0.01 vs. third bar, † *p* < 0.05 vs. fourth bar (Mann–Whitney U test). CM, conditioned medium; MC1R, melanocortin 1 receptor; MITF, microphthalmia-associated transcription factor; RAB27A, Ras-related protein Rab27A; RAB32, Ras-related protein Rab32; TYRP1, tyrosinase-related protein 1; TYRP2, tyrosinase-related protein 2; NVP, niacinamide + vitamin C + polydeoxyribonucleotide; UV, ultraviolet.

The expression levels of MC1R and MITF were increased by UV-CM treatment and decreased by NVP-mix treatment (Figure 3A,B). The expression levels of MC1R and MITF in the UV-CM group were not significantly different from those in the *si*NNT/UV-NVP-CM group. This result showed that the decreased expression levels of MC1R and MITF induced by UV-CM were not significantly different between normal and NNT-silenced melanocytes. The expression levels of MC1R and MITF were also decreased after UV-NVP-CM administration to NNT-silenced melanocytes, although this NVP-mix effect was not different between normal and NNT-silenced melanocytes.

The expression levels of TYRP1 and TYRP2 were increased by UV-CM treatment and decreased by NVP-mix treatment (Figure 3C,D). The increased expression levels of TYRP1 and TYRP2 induced by UV-CM were higher in NNT-silenced melanocytes than in normal melanocytes. NVP-mix did not significantly decrease the expression levels of TYRP1 and TYRP2 in NNT-silenced melanocytes.

The expression of RAB32 and RAB27A increased after UV-CM administration and decreased after NVP-mix administration (Figure 3E,F). The increased expression levels of RAB32 and RAB27A induced by UV-CM were significantly higher in NNT-silenced melanocytes than in normal melanocytes. The expression levels of RAB32 and RAB27A were also decreased by NVP-mix in NNT-silenced melanocytes; however, this effect was lower in NNT-silenced melanocytes than in normal melanocytes.

These results showed that NNT silencing led to increased expression of TYRP1, TYRP2, RAB32, and RAB27A after UV-CM treatment; however, the expression of MC1R and MITF after administration of UV-CM was not changed by silencing NNT. It is known that NNT induces tyrosinase degradation [21]. Since NNT directly affects tyrosinase, NNT silencing did not change the upstream pathway of tyrosinase, such as that which includes MC1R and MITF, in our study.

### 2.5. NVP-Mix Decreased Tyrosinase Activity and Melanin Deposition in Melanocytes and Animal Skin

**Figure 4:**
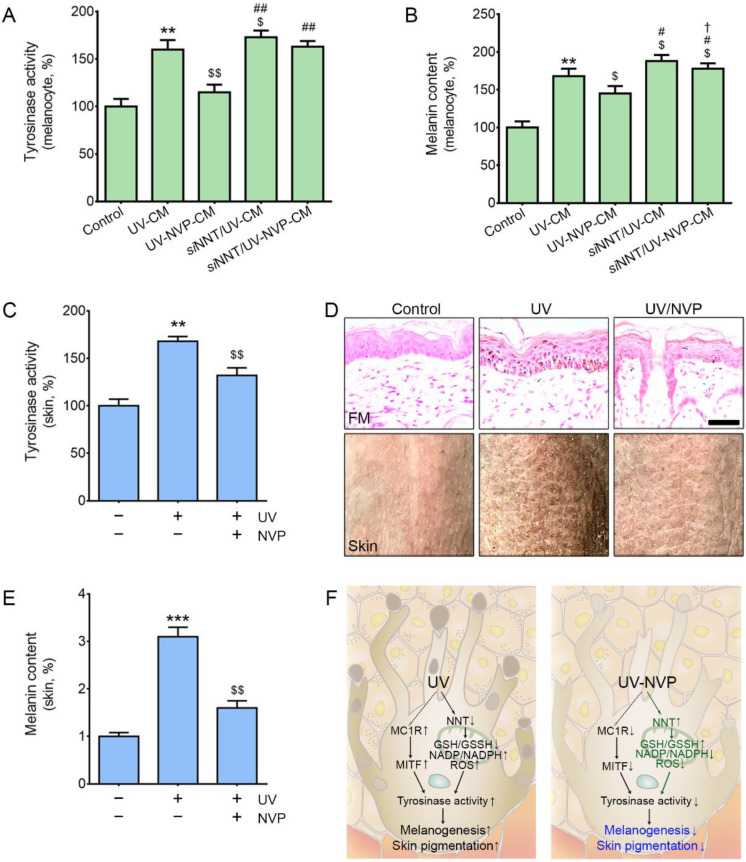
Regulation of tyrosinase activity and melanin content after NVP-mix treatment. (**A**,**B**) The tyrosinase activity (**A**) and melanin contents (**B**) were determined in CM-treated melanocytes. CM was obtained from UV-irradiated keratinocyte cultures treated with PBS or NVP-mix. (**C**) Tyrosinase activity was assessed in the UV-irradiated animal skin. (**D**,**E**) The melanin contents were assessed with Fontana–Masson staining in UV-irradiated animal skin (**D**, upper row scale bar = 100 µm). (**F**) Schematic summary of the effect of NVP-mix after UV irradiation. Data are presented as the mean ± standard deviation; ** *p* < 0.01, *** *p* < 0.001 second bar vs. first bar; $ *p* < 0.05, $$ *p* < 0.01 vs. second bar; # *p* < 0.05, ## *p* < 0.01 vs. third bar, † *p* < 0.05 vs. fourth bar (Mann–Whitney U test). CM, conditioned medium; FM, Fontana–Masson staining; GSH, glutathione; GSSG, oxidized glutathione; MC1R, melanocortin 1 receptor; MITF, microphthalmia-associated transcription factor; NADP^+^, nicotinamide adenine dinucleotide phosphate; NNT, nicotinamide nucleotide transhydrogenase; NVP, niacinamide + vitamin C + polydeoxyribonucleotide; ROS, reactive oxygen species; SOD, superoxide dismutase; UV, ultraviolet.

The tyrosinase activity was increased by UV-CM treatment and decreased by NVP-mix treatment (Figure 4A). The UV-CM-induced increase in tyrosinase activity in NNT-silenced melanocytes was significantly higher than that in normal melanocytes. In NNT-silenced melanocytes, NVP-mix did not decrease the tyrosinase activity that was increased by UV-CM.

The melanin content was increased by UV-CM and decreased by NVP-mix (Figure 4B). The increased melanin content induced by UV-CM in the NNT-silenced cells was significantly higher than that in normal melanocytes. In the NNT-silenced cells, NVP-mix decreased melanin content; however, this decrease was lower than that in normal melanocytes.

The tyrosinase activity in the UV-irradiated animal group was significantly higher than that in the control and UV/NVP animal groups (Figure 4C). The melanin content, which was evaluated by FM staining, was significantly higher in UV-treated mice than those in the control and UV/NVP groups (Figure 4D,E).

## 3. Discussion

The most well-known UV-induced melanogenesis signaling pathway is the increase in POMC in keratinocytes that leads to upregulation of MC1R/MITF/TYRP1 and TYRP2 in melanocytes [47]. A recently discovered signaling pathway involved in melanogenesis is the NNT-controlled direct modulation of tyrosinase activity [21]. NNT is involved in mitochondrial redox levels and leads to increased GSH contents in various tissues, such as the myocardium [48]. During the synthesis of pheomelanin, GSH or cysteine is needed [49,50]. Thus, NNT, which increases the level of GSH, could mediate pheomelanin synthesis rather than eumelanin synthesis [21]. In contrast, knockdown of NNT decreases the GSH/GSSG ratio, increases the NADP^+^/NADPH ratio and increases eumelanin synthesis in melanoma cells [21].

Nicotinamide, vitamin C, and PDRN are well-known antioxidants. Thus, we hypothesized that their combination (NVP-mix) could modulate redox levels by increasing NNT expression and decreasing mitochondrial oxidative stress to eventually decrease melanin synthesis mediated by the redox system. We therefore evaluated the ability of NVP-mix to decrease melanogenesis in UV-B-irradiated animal skin and in vitro in a human melanocyte model after treatment with CM from UV-B-irradiated keratinocyte cultures. For effective delivery of NVP-mix into the skin, a MTS was used in this study.

Microneedling can increase the efficacy of drug delivery by generating multiple microscopic channels in the skin made by microneedles [51,52]

The expression of NNT was significantly decreased by UV-B irradiation and increased by NVP-mix in UV-B-irradiated animal skin. The expression of NNT in the NVP-mix group was higher than that in the control group. Accompanied by the increased expression of NNT, the GSH/GSSG and NADPH/NADP^+^ ratios increased in UV-B-irradiated animal skin. Moreover, mitochondrial SOD activity was increased by NVP-mix. We also evaluated the effects of NVP-mix on NNT expression and oxidative stress levels in melanocytes treated with CM from UV-B-irradiated keratinocytes. The expression of NNT and the GSH/GSSG ratio in melanocytes both decreased after treatment with CM; however, they increased after NVP-mix administration. Additionally, the NADPH/NADP^+^ ratio was decreased by CM and increased by NVP-mix. SOD activity in the mitochondria was decreased by CM and increased by NVP-mix. These results suggested that NVP-mix controls the increase in oxidative stress in the mitochondria caused by UV-B irradiation.

To evaluate whether NVP-mix controlled oxidative stress via NNT, we treated NNT-silenced melanocytes with CM from UV-B-irradiated keratinocytes. When NNT was knocked down, the GSH/GSSG ratio was further decreased by CM than when compared with that observed in normal melanocytes. Additionally, NADPH/NADP^+^ ratio was likewise decreased by CM upon NNT knockdown. These results suggested that NNT is involved in controlling the GSH/GSSG and NADPH/NADP^+^ ratios and oxidative stress. NVP-mix also increased the GSH/GSSG and NADPH/NADP^+^ ratios even after NNT silencing; however, the level of change was lower than that found in normal melanocytes. Oxidative stress, which was evaluated by MitoSOX, also increased after treatment with CM from UV-B-irradiated keratinocytes; however, oxidative stress was decreased by NVP-mix and the latter effect was reduced after silencing NNT. These results showed that NVP-mix could control oxidative stress via NNT, although NNT is not the only factor that controls oxidative stress. NVP-mix might also influence other antioxidative pathways.

NVP-mix decreased melanogenesis signals, such as MC1R, MITF, TYRP1, and TYRP2, and decreased melanosome transfer-related signals, such as RAB32 and RAB27A, in UV-B irradiated skin. NVP-mix also decreased MC1R, MITF, TYRP1, TYRP2, RAB32, and RAB27A in melanocytes treated with CM from UV-irradiated keratinocytes. These results suggested that NVP-mix decreased melanogenesis-related signaling pathways. To evaluate whether NVP-mix decreased the melanogenesis signaling pathways via NNT, CM from UV-irradiated keratinocytes was used to treat NNT-silenced melanocytes. Here, the expression of MC1R and MITF was not changed by NNT silencing, but the expression of TYRP1, TYRP2, RAB32, and RAB27A was increased.

The ubiquitin–proteasome system is involved in tyrosinase degradation [53]. Overexpression of NNT leads to increased tyrosinase degradation; however, reversible proteasome inhibition leads to decreased tyrosinase degradation, which is induced by the overexpression of NNT [21]. After NNT silencing, MITF expression remained unchanged in melanocytes [21]. Thus, knockdown of NNT results in decreased tyrosinase degradation and increased tyrosinase stability [21]. Similar to this previously referenced report, our results showed that NNT silencing could not affect the CM-induced changes in the expression of MC1R or MITF, which are upstream of tyrosinase. NVP-mix decreased the expression levels of MC1R, MITF, RAB32, and RAB27A, which were increased by CM in NNT-silenced melanocytes. Notably, these decreases were lower than those in normal melanocytes. NVP-mix did not decrease the expression levels of TYRP1 or TYRP2, which were increased by CM in NNT-silenced melanocytes. The CM-induced enhancement in tyrosinase activity was also decreased by NVP-mix in normal melanocytes, but not in NNP-silenced melanocytes. These results suggested that NVP-mix could decrease tyrosinase activity by decreasing MITF as well as increasing NNT. In this study, we did not evaluate the protein level of tyrosinase by Western blotting, thus it was hard to confirm whether NNT was involved in degradation of tyrosinase. In future study, the exact mechanism of how NNT is involved in decreasing the activity of tyrosinase by NVP-mix should be evaluated.

Excessive skin pigmentation leads to cosmetic concerns, such as freckles, senile lentigines, and melasma [54,55]. Since tyrosinase activity is critical during melanogenesis, by which pathological local hyperpigmentation such as melasma and lentigo occur [54], decreasing tyrosinase activity by using tyrosinase inhibitors has been widely tried as a treatment for hyperpigmentation disorder [56,57,58,59,60,61]. Representative tyrosinase inhibitors are hydroquinone, kojic acid, azelaic acid, and electron-rich phenols, which have been used as therapeutics or cosmetics for hyperpigmentation [62]. However, hydroquinone leads to skin irritation and has possible mutagenic potential to mammalian cells [63,64]. Kojic acid and arbutin have poor efficacy in vivo due to their low-stability formulations and poor skin penetration [65]. It has also been reported that kojic acid shows carcinogenicity [66]. Thus, various natural products are preferred for these purposes because of their reduced adverse side effects and improved safety compared with synthetic products [67,68,69].

PDRN has been used for wound healing and has shown high safety [70]. Since the topical use of vitamin C and nicotinamide has also shown excellent safety [71,72], we thought that their combination (NVP-mix) could be used safely as a therapeutic for hyperpigmentation. NVP-mix decreased various signaling pathways, such as MITF, tyrosinase, TYRP1, and TYRP2 and reduced mitochondrial oxidative stress via NNT. Thus, through these mechanisms, NVP-mix could decrease skin pigmentation.

In conclusion, NVP-mix decreased mitochondrial oxidative stress by increasing NNT expression and decreased melanogenesis by decreasing the MC1R/MITF, tyrosinase, TYRP1, and TYRP2 signaling pathways.

## 4. Materials and Methods

### 4.1. Preparation of NVP-Mix

NVP-mix was formulated as a liquid prior to application. Niacinamide, vitamin C, and PDRN were dissolved in distilled water with mixing at 3000 rpm using a high-speed mixer (T.K. Homo Disper, Model 2.5, PRIMIX, Awaji-shi, Japan). Then, the NVP-mix solution was filtered through a 0.2 μm filter (S2GPU11RE, Merck, CA, USA) to remove bacteria. A total of 1 mM of NVP-mix liquid contains 0.55 mM of niacinamide, 0.25 mM of vitamin C, and 0.18 mM of PDRN [45].

### 4.2. UV-B-Radiated Mouse Model

HRM-2 mice (female, 6 weeks old, 20–25 g) were housed in cages with free access to food and water on a light-dark cycle of 12 h each at a controlled temperature of 22–25 °C.

After a 2-week stabilization period, the mice were randomly divided into three groups as follows: control, UV, and UV/NVP. The control group was treated with distilled water using a microlending treatment system (MTS; LARCO-STAMP-02; 16.5 cm high, 4.5 cm wide, 4.5 cm in the vertical direction, 0.75 mm needle length, 140 needles; L’arcobaleno, Seoul, Korea). The UV group was irradiated with UV-B for 5 min each time at an intensity of 200 mJ/cm^2^ for a total of 8 treatments in 13 days (once every 2 days for the first 10 days, then every day for the next 3 days) followed by administration of distilled water using the MTS. The UV/NVP group was irradiated with UV according to the same method as that used in the UV group and treated with NVP-mix using the MTS. After the appropriate treatment, the UV and UV/NVP groups were irradiated with UV-B at 2-day intervals and then treated with distilled water or NVP-mix using the MTS at 7-day intervals for 28 days. This study was approved by the Center of Animal Care and Use Ethics Board of Gachon University (approval number LCDI-2021-0068) and executed in accordance with the guidelines of the Institutional Animal Care and Use Committee.

### 4.3. In Vitro Model

#### 4.3.1. Cell Culture

Human primary epidermal keratinocytes (HEKn cells; cat. PCS-200-010, ATCC, Manassas, VA, USA) and human primary epidermal melanocytes (HEMn cells; cat. PCS-200-012, ATCC) were used. HEKn cells were cultivated with dermal cell basal medium (cat. cat. PCS-200-030, ATCC) with a keratinocyte growth kit (cat. PCS-200-040, ATCC), and HEMn cells were also cultivated with dermal cell basal medium with a melanocyte growth kit (cat. PCS-200-041, ATCC). All cells were maintained at 37 °C under 5% CO_2_.

#### 4.3.2. In Vitro Models and NNT shRNA Transfection

To determine whether melanocytes were affected by NVP-mix-treated keratinocytes, HEKn cells were irradiated with UV-B (200 mJ/cm^2^) for 5 min, treated with phosphate-buffered saline (PBS) or NVP-mix (1 mM), and cultured for 24 h. Then, the cell culture supernatants were collected (denoted as CMs) and treated with HEMn cells in the UV-CM and UV-NVP-CM groups, respectively. The NNT shRNA (cat. sc-150013-SH, Santa Cruz Biotechnology, Dallas, TX, USA; 1 µg) was transfected to HEMn cells using lipofectamine 3000 regent (Invitrogen, Waltham, MA, USA) for 12 h. After transfection, the cells were treated with the corresponding CM (*si*NNT/UV-CM and *si*NNT/UV-NVP-CM groups, respectively).

### 4.4. Sample Preparation

#### 4.4.1. Paraffin-Embedded Tissue

Skin tissues were harvested from the animals and fixed in cold 4% paraformaldehyde (cat. 16005, Sigma-Aldrich, St. Louis, MO, USA) dissolved in PBS for 24 h. After washing the fixed skin with tap water for 30 min, it was dehydrated and wax-infiltrated using a tissue processor (Tissue-Tek VIP^®^ 5 Jr, SAKURA Finetek, Tokyo, Japan) and then embedded using an embedding machine (Tissue-Tek^®^ TEC^TM^ 6, SAKURA Finetek) to form a paraffin block.

#### 4.4.2. Mitochondria Isolation

A mitochondria isolation kit was used to isolate the mitochondrial cells and tissue (cat. 89874, cat. 89801, Thermo Scientific, Waltham, MA, USA). The procedure was performed according to the manufacturer’s instructions.

#### 4.4.3. RNA Extraction and cDNA Synthesis

Total RNA from cells and frozen skins was extracted using RNAiso Plus (cat. 9109, Takara, Kyoto, Japan) according to the manufacturer’s instructions. The concentration of extracted RNA was determined with a Nanodrop 2000 spectrophotometer (Thermo Fisher Scientific). In addition, cDNA was synthesized from the extracted RNA using a PrimeScript First Strand cDNA Synthesis Kit (cat. 6110A, Takara) according to the manufacturer’s instructions.

### 4.5. Immunohistochemistry

Paraffin-embedded skin tissues were sectioned at 7 µm using a microtome (Leica, Wetzlar, Germany) and incubated overnight at 60 °C for slide attachment. Then, the sectioned slides were passed through a series of xylene and ethanol solutions (100%, 90%, 80%, 70%) to remove the paraffin and then hydrated with distilled water. The hydrated slides were incubated with citrate buffer (citrate-based, pH 6.0) using a microwave for 10 min for antigen retrieval. The tissue slides were washed with distilled water and PBS. To reduce nonspecific binding, the slides were incubated with M.O.M. reagent (cat. PK-2200, Vector Laboratories Inc., Burlingame, CA, USA) according to the manufacturer’s instructions. The blocked slides were incubated with primary antibody (NNT, 1:50, cat. sc-390236, Santa Cruz Biotechnology Inc., Dallas, TX, USA) for 12 h at 4 °C. The slides were then rinsed with PBS and incubated with a biotinylated secondary antibody (1:200, cat. BA-2000, Vector Laboratories Inc.) for 1 h at room temperature. The slides were again rinsed with PBS and then incubated with ABC reagent (cat. PK-4000, Vector Laboratories Inc.) according to the manufacturer’s instructions. After washing with PBS, the slides were developed for 3 min using a solution of 3,3′-diaminobenzidine tetrahydrochloride hydrate (cat. D5637, Sigma-Aldrich). Then, the slides were washed with PBS followed by distilled water and counterstained using hematoxylin solution (cat. S3309, DAKO, Glostrup Kommune, Denmark) for 1 min to better visualize the tissue morphology. After the slides were washed, they were dehydrated with absolute alcohol and mounted using xylene and dibutyl phthalate in xylene (DPX; cat. 06552, Sigma-Aldrich) for observation. Images of the stained slides were acquired with an optical microscope (Olympus Optical Co., Tokyo, Japan), and the intensity was analyzed using ImageJ software v.1.53s (NIH, Bethesda, MD, USA).

### 4.6. Enzyme-Linked Immunosorbent Assays (ELISAs)

The GSH/GSSG ratio (cat. V6611, Promega, Madison, WI, USA), NADPH/NADP^+^ ratio (cat. ab65349, Abcam, Waltham, MA, USA), SOD activity (cat. ab65354, Abcam) and tyrosinase activity (cat. ab252899, Abcam) in the mitochondria of CM-treated melanocytes and skin were measured using appropriate ELISA kits following the manufacturers’ instructions.

### 4.7. Immunocytochemistry

To measure the expression level of NNT in CM-treated melanocytes, HEMn cells were seeded at 3 × 10^5^ cells/well in 8-well chamber slides (cat. 54534, Nunc, Waltham, MA, USA) for 12 h. After applying CM to the HEMn cells for 24 h at 37 °C under 5% CO_2_, the cells were washed with PBS, fixed with 100% methanol for 5 min at room temperature and washed with PBS again. The washed cells were treated with normal serum for 1 h at room temperature to reduce nonspecific antigen–antibody interactions. Then, the cells were incubated with primary antibody (NNT, 1:50, cat. sc-390236, Santa Cruz Biotechnology Inc., Dallas, TX, USA) for 12 h at 4 °C. The cells were rinsed with PBS and then incubated with a secondary antibody (1:500, Alexa Fluor 488; cat. A110029, Invitrogen, Waltham, MA, USA) for 1 h at room temperature. The cells were again rinsed with PBS and stained with DAPI (1 mg/mL, 1:1000, cat. D9542, Sigma-Aldrich, Burlington, MA, USA) for 10 sec at room temperature for nuclear visualization. After washing, the cells were mounted using vector shield solution (cat. H-1000, Vector Laboratories Inc.) for observation. Images of the stained cells were acquired with a confocal microscope (LSM 710, Carl Zeiss, Oberkochen, Germany), and the images were analyzed with Zen 2009 software (Carl Zeiss).

### 4.8. MitoSOX Red Staining

To quantify the mitochondrial superoxide production in CM-treated melanocytes, the cells were stained with MitoSOX Red (cat. M36008, Invitrogen). HEMn cells (0.6 × 10^4^ cells/well) were seeded into 24-well culture plates. After applying CM to the cells for 24 h at 37 °C under 5% CO_2_, the cells were washed with PBS. The washed cells were treated with 5 µM MitoSOX Red for 30 min at 37 °C. The cells were again washed with PBS, and the nuclei were stained with Hoechst 33342 (cat. H 3570, Invitrogen). Florescence images were obtained using a microscope (LSM-700) at Core-Facility for Cell to In-Vivo imaging (Incheon, Korea), and analyzed with ImageJ software (NIH).

### 4.9. Quantitative Real-Time Polymerase Chain Reaction (qRT–PCR)

The qRT–PCR reagent mixture was prepared by mixing 1 µg of synthesized cDNA, SYBR Green reagent (cat. RR82LR, Takara), and 10 pmol of primer (Supplementary Table S1). This mixture was added to a 384-well multiplate and analyzed with a CFX386 Touch Real-Time PCR System (Bio-Rad, Hercules, CA, USA).

### 4.10. Melanin Content Assays

To assess the melanin content in HEMn cells, CMs were applied to the cells for 24 h at 37 °C under 5% CO_2_. Then, the cells were collected by trypsin-EDTA and centrifuged at 12,000× *g* for 20 min. After centrifugation, the supernatants were discarded, and the pellets were dissolved in 100 µL of 10% dimethyl sulfoxide and 1 N NaOH solution at 95 °C for 20 min. Then, the cells were plated into 96-well plates, and the absorbance of each well was measured at 490 nm using a microplate reader (Molecular Devices, San Jose, CA, USA).

### 4.11. Fontana-Masson (FM) Staining

Paraffin-embedded tissue slides were stained with a Fontana-Masson Stain Kit (Scytek, Logan, UT, USA; FMS-1-IFU) for melanin visualization. The slides were incubated overnight at 60 °C for slide attachment. Then, the sectioned slides were passed through a series of xylene and ethanol solutions (100%, 90%, 80%, 70%) to remove the paraffin and then hydrated with distilled water. The hydrated slides were incubated in warmed ammoniacal silver solution for 30 min at 60 °C, rinsed with distilled water 3 times, and incubated in 0.2% gold chloride solution for 30 sec at room temperature. After the slides were washed with distilled water, they were incubated in 5% sodium thiosulfate solution for 1 min at room temperature and rinsed with running tap water and distilled water. To stain the nuclei, the slides were incubated with nuclear fast red stain for 5 min at room temperature. After washing, the slides were incubated with dehydrated absolute alcohol and mounted using xylene and DPX for observation. Images of the stained slides were acquired with an optical microscope (Olympus Optical Co., Tokyo, Japan) and the intensity was analyzed using ImageJ software (NIH, Bethesda, MD, USA).

### 4.12. Statistical Analysis

All results are presented as the means ± standard deviations, and all statistical analyses were performed by using SPSS version 22 (IBM Corporation; Armonk, NY, USA). Statistical significance was determined by the Kruskal–Wallis test for comparisons of each group, followed by a post hoc Mann–Whitney U test. In this study, groups marked with different letters indicate significant intergroup differences.

## Data Availability

All data are contained within the article.

## Supplementary Materials

The following supporting information can be downloaded at: https://www.mdpi.com/article/10.3390/molecules27154923/s1, Table S1: List of primers used for quantitative real-time polymerase chain reaction. Figure S1: Schematic diagram of the in vitro experiment in this study. Figure S2: Regulation of the expression of MC1R, MITF, TYRP1, TYRP2, RAB32, and RAB27A after NVP-mix treatment of UV-irradiated animal skin.

## Sample Availability

Sample of the compounds are not available from the authors.
