# Supplementary material for: The Combination of Niacinamide, Vitamin C, and PDRN Mitigates Melanogenesis by Modulating Nicotinamide Nucleotide Transhydrogenase
Source: Molecules. 2022 Aug 2;27(15):4923. doi: 10.3390/molecules27154923 (PMC9370691; doi:10.3390/molecules27154923)
Supplement: Supplementary file 1 [file molecules-27-04923-s001.zip › molecules-1828706-supplementary -final.pdf]

molecules

Supplementary Materials

The Combination of Niacinamide, Vitamin C, and PDRN Mitigates Melanogenesis by Modulating Nicotinamide Nucleotide Transhydrogenase

Hyun Jun Park[1,†], Kyung-A Byun[2,3,†], Seyeon Oh[3], Hyoung Moon Kim[2], Moon Suk Chung[4], Kuk Hui Son[5,*], Kyunghee Byun[2,3,*]

[1] Maylin Anti-Aging Center Apgujeong, Seoul 06005, Korea; parmani@naver.com (H.J.P.)
[2] Department of Anatomy & Cell Biology, College of Medicine, Gachon University, Incheon 21936, Korea; kabyun95@gmail.com (K.-A.B.); drmac12@me.com (H.M.K.)
[3] Functional Cellular Networks Laboratory, Graduate School and Lee Gil Ya Cancer and Diabetes Institute, College of Medicine, Gachon University, Incheon 21999, Korea; seyeon8965@gmail.com (S.O.)
[4] I`ll Global Co., Inc., Seoul 06532, Korea; sugi0821@empas.com (M.S.C.)
[5] Department of Thoracic and Cardiovascular Surgery, Gil Medical Center, Gachon University, Incheon 21565, Korea
* Correspondence: dr632@gilhospital.com (K.H.S.); khbyun1@gachon.ac.kr (K.B.); Tel.: +82-32-460-3666 (K.H.S.); +82-32-899-6511 (K.B.)
† These authors have contributed equally to this work.

Citation: Park, H.J.; Byun, K.-A.; Oh, S.; Kim, H.M.; Chung, M.S.; Son, K.H.; Byun, K. The combination of niacinamide, vitamin C, and PDRN mitigates melanogenesis by modulating nicotinamide nucleotide transhydrogenase. *Molecules* **2022**, *27*, 4923. https://doi.org/10.3390/molecules27154923

Academic Editor: Alejandro Samhan-Arias

Received: 6 July 2022
Accepted: 30 July 2022
Published: 2 August 2022

Publisher's Note: MDPI stays neutral with regard to jurisdictional claims in published maps and institutional affiliations.

Copyright: © 2022 by the authors. Submitted for possible open access publication under the terms and conditions of the Creative Commons Attribution (CC BY) license (https://creativecommons.org/licenses/by/4.0/).

Table S1. List of primers used for quantitative real-time polymerase chain reaction.

Gene (Host)		Primer
Actb (Mouse)	Forward	5'-CCG TAA AGA CCT CTA TGC CAA C-3′
	Reverse	5'-GCA GTA ATC TCC TTC TGC ATC C-3′
MC1R (Mouse)	Forward	5'-CAA GCA GCG GCA GAA AAA GT-3′
	Reverse	5'-TGG GAC TCA GAT ACG CCTGA-3′
MITF (Mouse)	Forward	5'-GAG AAC TGC AGC CAG GAA CT-3′
	Reverse	5'-GCC GCA TTT AGA AAG CGA GA-3′
TYRP1 (Mouse)	Forward	5'-AGG GTG GAC CAA TCA GGA GA-3′
	Reverse	5'-CCG CAT CAG TGA AAG TGT GC-3′
TYRP2 (Mouse)	Forward	5'-CCT GAA TGG GAC CAA TGC CT-3′
	Reverse	5'-GAA AAG CCA GCA ACC CCA AG-3′
RAB32 (Mouse)	Forward	5'-GAC AGG AAC GGT TTG GCA AC-3′
	Reverse	5'-GGG TAA AGT CAC AGG GCC TC-3′
RAB27A (Mouse)	Forward	5'-GTG GGG CCA GAC GGA AAA TA-3′
	Reverse	5'-GTC CTC GCT GTG CTC TAT CC-3′
Actb (Human)	Forward	5'-CTC GCC TTT GCC GAT CC-3′
	Reverse	5'-TCT CCA TGT CGT CCC AGT TG-3′
MC1R (Human)	Forward	5'-ACT TCT CAC CAG CAG TCG TG-3′
	Reverse	5'-CAT TGG AGC AGA CGG AGT GT-3′
MITF (Human)	Forward	5'-AGA GAA GGT GCA CGT AAG CG-3′
	Reverse	5'-ATC TGC ATA CAG GAC GCT CG-3′
TYRP1 (Human)	Forward	5'-GTG CCA CTG TTG AGG CTT TG-3′
	Reverse	5'-ATG GGG ATA CTG AGG GCT GT-3′
TYRP2 (Human)	Forward	5'-AAA GCC TGA CTT AAC GGG GG-3′
	Reverse	5'-GGA TTT TGC AGC CCA AGC AA-3′
RAB32 (Human)	Forward	5'-TCC AAA TGG CAG CCC TAT CC-3′
	Reverse	5'-CAT CCG GCA AAG CCA TGT TC-3′
RAB27A (Human)	Forward	5'-AGT TGA TGG AGC GAA CTG CT-3′
	Reverse	5'-CCC TAC ACC AGA GTC TCC CA-3′

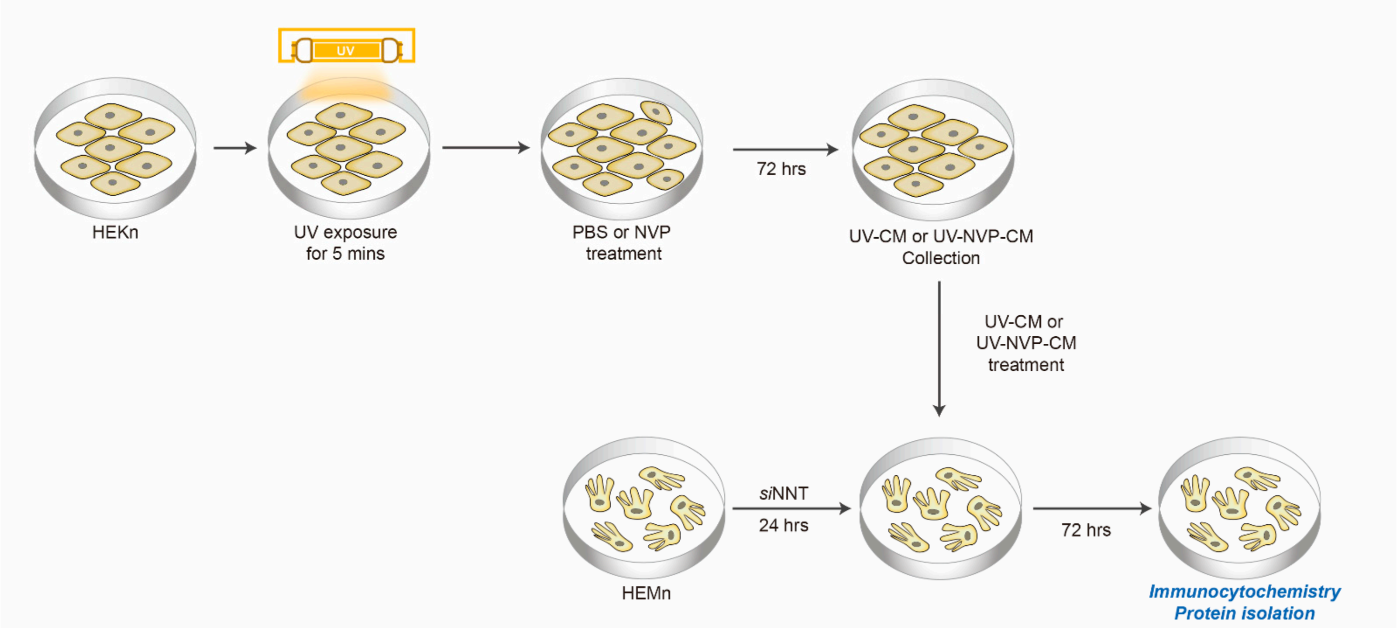

Figure S1. Schematic diagram of the in vitro experiment in this study. Human keratinocytes were exposed to UV irradiation for 5 mins, and then NVP-mix was added. The supernatants from these cell cultures were collected (CMs). Then, melanocytes or NNT-silenced melanocytes were treated with CM. CM, conditioned medium; HEKn; human primary epidermal keratinocyte; HEMn; human primary melanocyte; hrs, hours; mins, minutes; NVP, niacinamide + vitamin C + polydeoxyribonucleotide; PBS, phosphate-buffered saline; *si*NNT, silencing with nicotinamide nucleotide transhydrogenase; UV, ultraviolet.

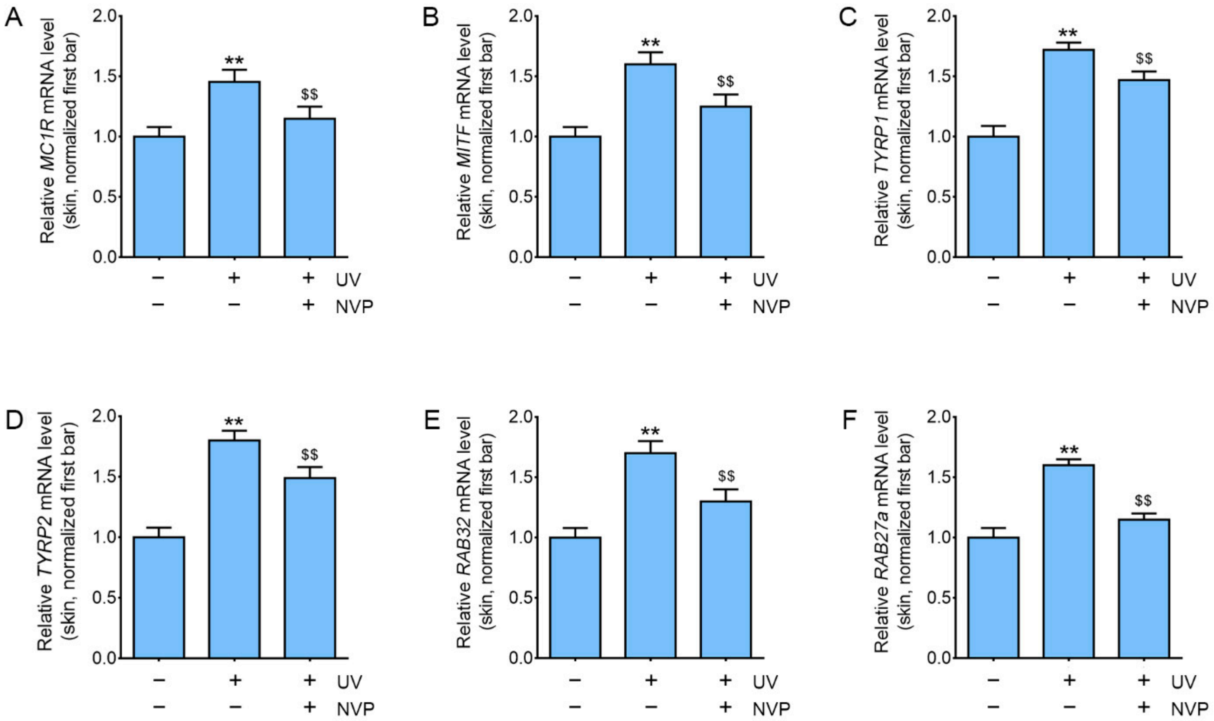

Figure S2. Regulation of the expression of MC1R, MITF, TYRP1, TYRP2, RAB32, and RAB27A after NVP-mix treatment of UV-irradiated animal skin. (A–F) The mRNA expression levels of MC1R (A), MITF (B), TYRP1 (C), TYRP2 (D), RAB32 (E), and RAB27A (F) determined by quantitative real-time polymerase chain reaction in UV-irradiated animal skin. The mRNA levels were normalized to that of Actb and are expressed relative to the corresponding level in the control group. Data are presented as the mean ± standard deviation; **, p < 0.01 second bar vs. first bar; $, p < 0.05, $$, p < 0.01 vs. second bar (Mann–Whitney U test). MC1R, melanocortin 1 receptor; MITF, microphthalmia-associated transcription factor; RAB27A, Ras-related protein Rab27A; RAB32, Ras-related protein Rab32; TYRP1, tyrosinase-related protein 1; TYRP2, tyrosinase-related protein 2; NVP, niacinamide + vitamin C + polydeoxyribonucleotide; UV, ultraviolet.
